# Relationship between workplace violence and mental/physical health of security guards during the COVID-19 pandemic in Taiwan

**DOI:** 10.3389/fpubh.2024.1333139

**Published:** 2024-02-26

**Authors:** Ying-Han Lee, Yun-Hsuan Wu, Chiu-Ying Chen, Patricia Chiao-Tzu Lee, Tzu-Hsien Lin, Chane-Yu Lai

**Affiliations:** ^1^Department of Public Health, China Medical University, Taichung, Taiwan; ^2^School of Medicine and Dentistry, Gold Coast Campus, Griffith University, Southport, QLD, Australia; ^3^Department of Occupational Safety and Health, Chung Shan Medical University, Taichung, Taiwan

**Keywords:** workplace violence, security guards, mental and physical health, COVID-19, CHQ-12

## Abstract

**Objectives:**

To investigate the relationship between workplace violence (WPV) and mental and physical health (MPH) of security guards during the COVID-19 pandemic in Taiwan.

**Methods:**

A cross-sectional survey was conducted in 15 representative security companies across northern, central, and southern Taiwan, and outlying islands from July 2021 to June 2022 during the COVID-19 pandemic. 1,200 questionnaires were distributed. A total of 1,032 valid questionnaires were collected.

**Results:**

13.18% of the participants reported that they had experienced WPV during the COVID-19 pandemic, including physical violence (PhV), psychological violence (PsV), verbal violence (VV), and sexual harassment (SH). The most common violence was VV (54.19%), followed by PsV (20.69%). Community residents and property owners were the primary perpetrators, followed by strangers. The study showed that the security guards who had experienced WPV had higher scores on the 12-item Chinese Health Questionnaire (Taiwan version) (CHQ-12), indicating poorer MPH than those who had never experienced WPV. The result showed that VV had strong correlations with the lack of effective communication, dissatisfaction with treatment and service attitude, and work stress. PsV was strongly associated with excessive waiting times.

**Conclusion:**

There were correlations among PhV, VV, and PsV and they had adverse impacts on MPH, except for SH. The study found that the primary perpetrators of WPV against security guards were community residents and property owners. The causes were the lack of effective communication, dissatisfaction with treatment and service attitude, excessive waiting times, and work stress, which further led to turnover intention and poor MPH. The findings of this study have useful implications and it is recommended to enhance the understanding of workplace violence against security guards and to formulate appropriate local and international strategies to address it.

## 1 Introduction

The main responsibilities of security guards are to watch over the premises, manage visitor entries at the gates, protect the stationed locations, safeguard any ongoing events, and traffic control. Since the COVID-19 outbreak, security guards had to conduct several additional duties such as greeting the visitors with hand sanitizer and temperature measurement gun, instructing visitors to sanitize their hands, wear masks, and forbidding entry to people who did not comply to the rules (1). In 2022, there were 90,000 current employees in the security industry in Taiwan; the majority was static security guards, comprising around 80%. Security guards not only needed to carry out regular job duties, but were also required to undertake additional preventive measures as per government guidelines during the COVID-19 pandemic. Meanwhile, they were not granted any legal authority, leaving them to experience frequent incidents of workplace violence (2).

During the pandemic, medical professionals, police officers, and security guards had to be on the front line of epidemic prevention, facing rampant epidemics, increased workload, work stress, and therefore their physical and mental health (MPH) were negatively affected during this period of time. (3, 4). A systematic review in the United States (U.S.) that COVID-19 was a catalyst for the occurrence of Workplace violence (WPV) (5, 6). The workers were undergoing a deterioration of mental health caused by the increased WPV (7). Previous epidemiological research found that workers who have experienced WPV or workplace bullying tend to experience a higher risk of mental and physical diseases, including anxiety, depression (8–12), and sleep problems (13, 14).

According to U.S. National Crime Victimization Survey (15), private security work was rated as the third-most violence-prone occupation after bartenders and law enforcement officers. A Finnish study estimated monthly prevalence rates of security guards were 39% for verbal aggression, 19% for threats of assault, and 15% for physical acts (16). Additionally, French security guards reported a 40% of exposure rate for physical and verbal violence (17). Moreover, in 2014, a Taiwanese government agency surveyed on workplace violence among employees of 30 occupations and found that security workers experienced the highest prevalence rate of 16.7% for any form of violence (8). However, a study in the United States showed that violence against security guards dropped from 20 to 11 cases within 4 months in hospitals, and from 13 to 4 cases in the emergency departments (ED), respectively during the COVID-19 period (18). It showed that the WPV against security personnel during the pandemic needed further investigation.

Workplace violence is a significant concern in public health and occupational safety. The International Labor Organization (ILO) and the World Health Organization (WHO) define workplace violence as: “Incidents where staff are abused, threatened or assaulted in circumstances related to their work, involving an explicit or implicit challenge to their safety, well-being or health” (19). Physical violence (PhV) involves intentional use of violence, in which the aggressor acts against an individual or a group, leading to physical, sexual, or psychological harm. Verbal violence (VV) causes harm to the victim through direct or indirect means, whether verbally or in writing. Psychological Violence (PsV) means different forms of malicious behaviors. Victims are often afraid or unable to resist effectively due to power inequality and end up feeling threatened, humiliated or embarrassed. Sexual harassment (SH) is defined as any unwanted, unreciprocated, and unwelcome behavior of a sexual nature that is offensive to the individual involved, resulting in feelings of threat, humiliation, or embarrassment (20). According to the ILO, WPV perpetrated between coworkers is defined as “horizontal violence”; while WPV that takes place between supervisors and subordinates is “vertical violence”. In addition, WPV caused by customers, residents, patients, or the public is defined as “third party violence” (21).

The aim of this study is to investigate WPV experienced by security guards during COVID-19 pandemic, including the WPV types, primary perpetrators, relevant factors, and the relationship between MPH. The results of this research study hold useful implications which can be employed as a reference for the government, academic institutions, and relevant agencies in developing policies to prevent workplace violence.

## 2 Materials and methods

### 2.1 Research design and research subjects

Due to the limitations imposed by the COVID-19 pandemic, this study conducted a cross-sectional survey and adopted purposive sampling among the 2,477 employees who were willing to participate in the survey from 15 security companies across northern, central, and, southern, the outlying islands of Taiwan. Data were collected anonymously by distributing hard-copy questionnaires (22). The categories of security guards included system, static, private, and patrol security. However, supervisors, such as the general manager, vice-general manager, deputy director, team leaders, and unpaid/absent employees were excluded.

This study used questionnaire of “Study on Industries with High-Risk Workplace Violence and Preventive Strategies” conducted by the Institution of Labor, Occupational Safety and Health, Ministry of Labor, Taiwan (23), which was based on the questionnaire by ILO/ICN/WHO/PSI. The content validity was reviewed by an expert committee, consisted of one security guard manger and four principal investigators. Those are expert and experiences on Workplace Violence field in Taiwan. Then, the internal reliability was measured by pre-test and post-test done in 30 participants. This study has been approved by the Behavioral and Social Science Research Ethics Committee, China Medical University Hospital (Identifier: CMUH110-REC1-124); the survey time frame spanned from July, 2021 to June, 2022.

The sample size was determined using G-power software 3.1 (24), the F-test multiple regression analysis was conducted to analyze the prediction of questionnaires, which resulted in a coefficient of determination *R*^2^ = 0.29, indicating the explanatory power of this study, with an effect size *f*^2^ = 0.408, error probability *α* = 0.05, a power (Ι-β error probability) of 0.8, and 200 number of predictors. The calculation indicated that a sample size of 286 participants would be sufficient. However, after considering response rate and the desire to enhance the accuracy of statistical inference, the researchers opted to distribute 1,200 participants. 1,133 participants were collected, and the response rate was 94%. Invalid questionnaires were identified due to missing information. Eventually, 101 invalid questionnaires were excluded. There were 1,032 participants in the final analysis and net response rate was 86%.

### 2.2 Measurements of key variables

The questionnaire content consisted of closed-ended and open-ended questions and was divided into six sections. The first section is social demographic information, including sex, age, education level, and medical records. Also, there are employment-related variables, including tenure in current position and categories of job duties. In some survey questions, we include “others” and open-ended questions as an option for participants to fill out detailed information. The second to the fifth sections pertained to PhV, VV, PsV, and SH. In these sections, we asked respondents questions like: “In the last 12 months, have you experienced physical violence (verbal violence, psychological violence, and sexual harassment) at your workplace?” The responses were “yes” or “no.” These sections included questions regarding the frequency of WPV in the previous year, perceptions about the occurrences of WPV, the perpetrators, locations, reasons, and responses to the incidents.

In the sixth section, we used the 12-item Chinese Health Questionnaire (CHQ-12) revised in 1986 to measure the participants’ MPH (25). There are 12 self-administered questions with responses using four-point scale: from “not at all,” “same as usual,” “rather more than usual,” to “much more than usual.” Questions from 1 to 10 were positively worded, while questions 11 and 12 were negatively worded, with a total score ranging from 0 to 12. The options for negative were assigned 0-0-1-1, respectively, and the corresponding marks for positive ones were reversed (26). In addition, the 12 questions were categorized into four facets: questions 1–4 addressed somatization symptoms; questions 6, 9, and 11 focused on anxiety and worry; questions 7, 8, 10, and 12 examined depression or poor family relationships, while question 5 specifically targeted sleep disorders (27, 28). In this study, a score of 5 represented the cut-off point, and the scores greater than or equal to 5 meant poor MPH (29, 30). Before 1,200 questionnaires were distributed, we conducted a pre-test and post-test of 30 questionnaires. The reliability scale, as measured by Cronbach’s alpha = 0.85. Overall, in the case of missing items (up to one-third missing items allowed), the total score is calculated by multiplying the mode of the responses by the number of missing answer.

### 2.3 Statistical analysis

This study adopted SAS version 9.4 statistical software to analyze the data. Descriptive statistical analysis was used to present the distributions of demographic characteristics and WPV prevalence. Cronbach’s alpha was used to measure the reliability of different scales and assess the internal consistency of these scales. Multiple logistic regression was conducted to investigate the risk factors of WPV and the relationship between WVP experience and MPH of the security guards. Moreover, Wilcoxon’s rank-sum test was employed to present the differences between the MPH of the security guards who had experienced WPV versus those who had not.

## 3 Results

Table 1 showed the distribution of demographic variables, categories of job duties, job tenure, medical records, and WPV experience. The majority of participants were males (92.00%). The average age of the male participants ranged from 31 to 40 years old (27.61%). Most of the participants received high school or vocational school education (46.99%). There were 637 (61.72%) static, 132 (12.79%) private, 96 (9.3%) systematic, 80 (7.75%) patrol security guards, and 87 (8.43) others. Static security guards took up the highest percentage of participants. The rest were administrative staff, totaling 87 (8.43%). A total of 39.41% of the participants had been working for 1–3 years. There were 199 (20.97%) participants having medical records. Table 1 presented the types of WPV. In our sample, the prevalence of participants’ experiences with different types of violence in the preceding 12 months were: PhV (3.59%), VV (10.66%), PsV (4.07%), and SH (0.36%), respectively. A total of 89 participants scored higher than or equal to 5 in CHQ-12 (8.62%) and 943 participants scored lower than 5 (91.38%).

**Table 1:** Distribution of demographic variables of participants.

Variables	Total (*n* = 1,032)		Male (*n* = 949, 92%)		Female (*n* = 83, 8.0%)	
	*n*/mean	(%)SD	*n*/mean	(%) SD	*n*/mean	(%)SD
Age						
≦30	101	9.79	81	8.54	20	24.10
31–40	280	27.13	262	27.61	18	21.69
41–50	273	26.45	257	27.08	16	19.28
51–60	267	25.87	247	26.03	20	24.10
≧60	111	10.76	102	10.75	9	10.84
Education level						
Junior high and under	55	5.33	49	5.16	6	7.23
High/Vocational school	669	64.83	630	66.39	39	46.99
College and above	308	29.84	270	28.45	38	45.78
Position category						
System security	96	9.30	87	9.17	9	10.84
Static security	637	61.72	605	63.75	32	38.55
Private security	132	12.79	127	13.38	5	6.02
Patrol security	80	7.75	79	8.32	1	1.20
Others	87	8.43	51	5.37	36	43.37
Tenure, year						
Under 1	148	14.34	128	13.49	20	24.10
1–3	414	40.12	374	39.41	40	48.19
3–5	283	27.42	269	28.35	14	16.87
5–10	109	10.56	102	10.75	7	8.43
≧10	78	7.56	76	8.01	2	2.41
Medical record						
None	818	79.26	750	79.03	68	81.93
Yes	214	20.74	199	20.97	15	18.07
WPV						
None	896	86.82	828	87.25	68	81.93
PhV	37	3.59	34	3.58	3	3.61
VV	110	10.66	99	10.43	11	13.25
PsV	42	4.07	38	4.00	4	4.82
SH	14	1.36	9	0.95	5	6.02
CHQ-12						
<5	943	91.38	871	91.78	72	86.75
≧5	89	8.62	78	8.22	11	13.25

Table 2 presents the relationships between various types of WPV and MPH. The results showed that *r* = 0.46 (*p* < 0.001) for physical with verbal violence, *r* = 0.28 (*p* < 0.001) for physical with psychological violence, and *r* = 0.29 (*p* < 0.001) for physical with sexual harassment. Additionally, significant correlations with *r* = 0.36 (*p* < 0.001) for verbal violence and psychological violence and *r* = 0.18 (*p* < 0.001) for verbal violence and sexual harassment were identified. A moderate correlation between psychological violence and sexual harassment was also found, with *r* = 0.23 (*p* < 0.001). Moreover, we conducted a correlation analysis between PHV, VV, PSV, and SH, with CHQ-12 (MPH) total scores, obtaining correlation coefficients of 0.09 (*p* < 0.01), 0.18 (*p* < 0.0001), 0.25 (*p* < 0.0001), and 0.03 (*p* > 0.05), respectively. The results showed that all forms of WPV were significantly associated with MPH (*p* < 0.05), except for sexual harassment *r* = 0.03 (*p* > 0.05), which did not reach a statistically significant correlation.

**Table 2:** The bivariate correlation between various types of WPV.

Variables	PhV	VV	PsV	SH	CHQ
PhV	1				
VV	0.46^***^	1			
PsV	0.28^***^	0.36^***^	1		
SH	0.29^***^	0.18^***^	0.23^***^	1	
CHQ	0.09^**^	0.18^***^	0.25^***^	0.03	1

^*^p<0.05; ^**^p<0.01; and ^***^p<0.001.

Table 3 presented the distribution of the factors which affected MPH. This research indicates the primary source of VV and PsV were third-party perpetrators, comprising 81.82 and 64.29%, followed by horizontal violence (10.00 and 19.05%) and vertical violence (8.18 and 16.67%). There was a statistically significant correlation between MPH among those exposed to verbal violence and vertical violence (from supervisors/subordinates). VV and PsV were the primary WPV, which resulted from the lack of effective communication (46.79 and 57.14%), followed by attitude problems (32.11 and 57.14%). The contributing factors for VV and PsV reported by the security guards were organizational reform (10.09 and 14.29%), workload (26.61 and 45.24%), and work stress (51.38 and 45.24%). It was found that among those who experienced VV, the main reason for their poorer MPH was work stress (*p* < 0.05). The main behavioral reaction after experiencing VV and PsV was seeking colleagues’ assistance (41.82 and 33.33%), followed by burnout management (24.55 and 33.33%) and conceiving turnover intention (14.55 and 14.29%). Among them, the turnover intention of VV and PsV had a significant correlation with MPH (*p* < 0.05).

**Table 3:** The distribution and analysis of factors affecting the MPH of participants who had experienced VV and PsV, and their behavioral reactions.

Variables	CHQ-12 (≧5 score)					
	VV (*n* = 110)			PsV (*n* = 42)		
	*n*	%	*p*	*n*	%	*p*
Perpetrators						
Horizontal (from colleagues)	11	(10.00)	-	8	(19.05)	-
Vertical (from supervisors/subordinates)	9	(8.18)	^*^	7	(16.67)	-
Third-party violence (clients/third part)	90	(81.82)	-	27	(64.29)	-
Causes						
Long waiting times	25	(22.94)	-	4	(9.52)	^*^
Miscommunication	51	(46.79)	^*^	24	(57.14)	-
Mismanagement	19	(17.43)	^*^	9	(21.43)	-
Attitude problems	35	(32.11)	-	24	(57.14)	-
Excessive force	10	(9.17)	-	3	(7.14)	-
Interest conflict	8	(7.34)	-	3	(7.14)	-
Personal reason	19	(17.43)	-	9	(21.43)	-
Others	19	(17.43)	-	7	(16.67)	-
Implicit factors						
Organizational reform	11	(10.09)	-	6	(14.29)	-
Workload	29	(26.61)	-	19	(45.24)	-
Work stress	56	(51.38)	^*^	19	(45.24)	-
Social insecurity	34	(31.19)	-	9	(21.43)	-
Deterioration of interpersonal relationship	36	(33.03)	-	13	(30.95)	-
Response						
Burnout management	27	(24.55)	-	14	(33.33)	-
Verbal warning	15	(13.64)	-	3	(7.14)	-
Legal action	2	(1.82)	-	1	(2.38)	-
Colleagues’ assistance	46	(41.82)	-	14	(33.33)	-
Psychological counseling	-	-	-	1	(2.38)	-
Position transfer	4	(3.64)	-	3	(7.14)	-
Turnover intention	16	(14.55)	^***^	6	(14.29)	^*^

-, not significant; ^*^p < 0.05; ^**^p < 0.01; and ^***^p < 0.001.

**Figure 1:**
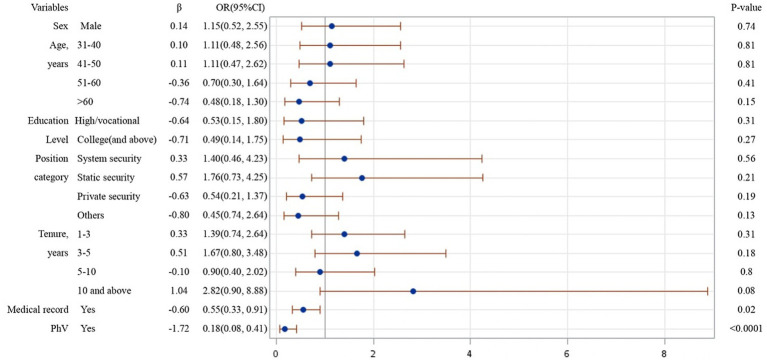
Logistic regression analysis on risk factors affecting MPH of participants experienced PhV.

**Figure 2:**
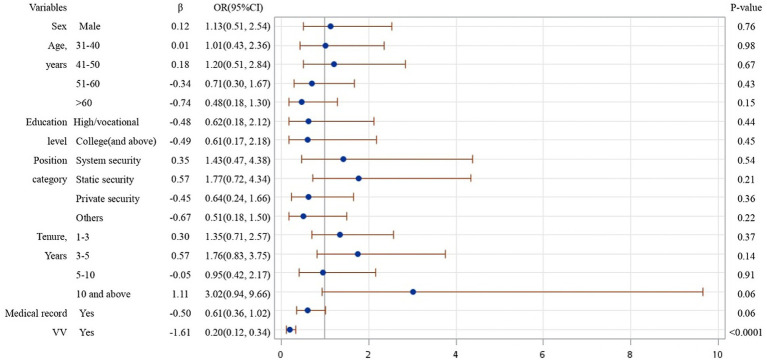
Logistic regression analysis on risk factors affecting MPH of participants experienced VV.

**Figure 3:**
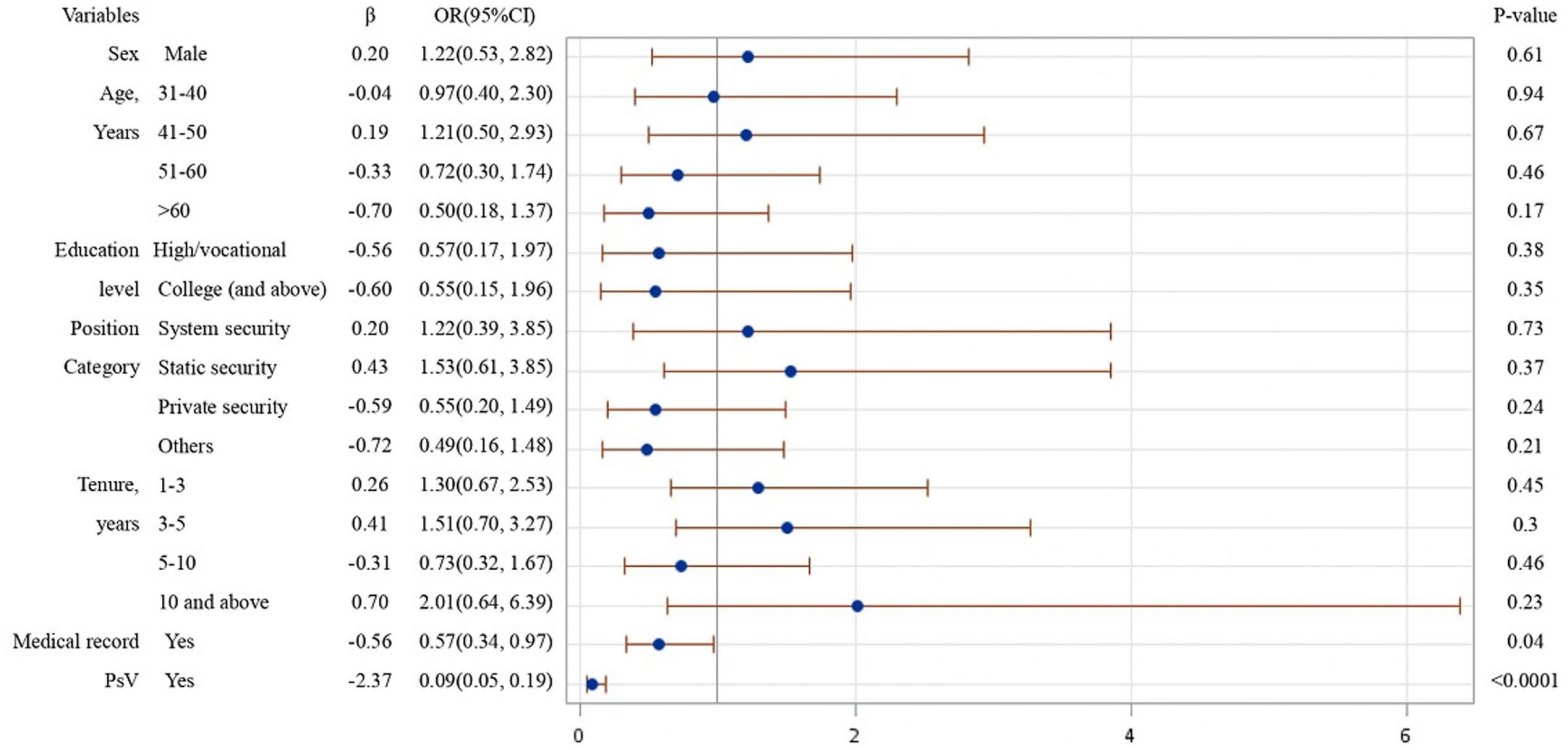
Logistic regression analysis on risk factors affecting MPH of participants experienced PsV.

**Figure 4:**
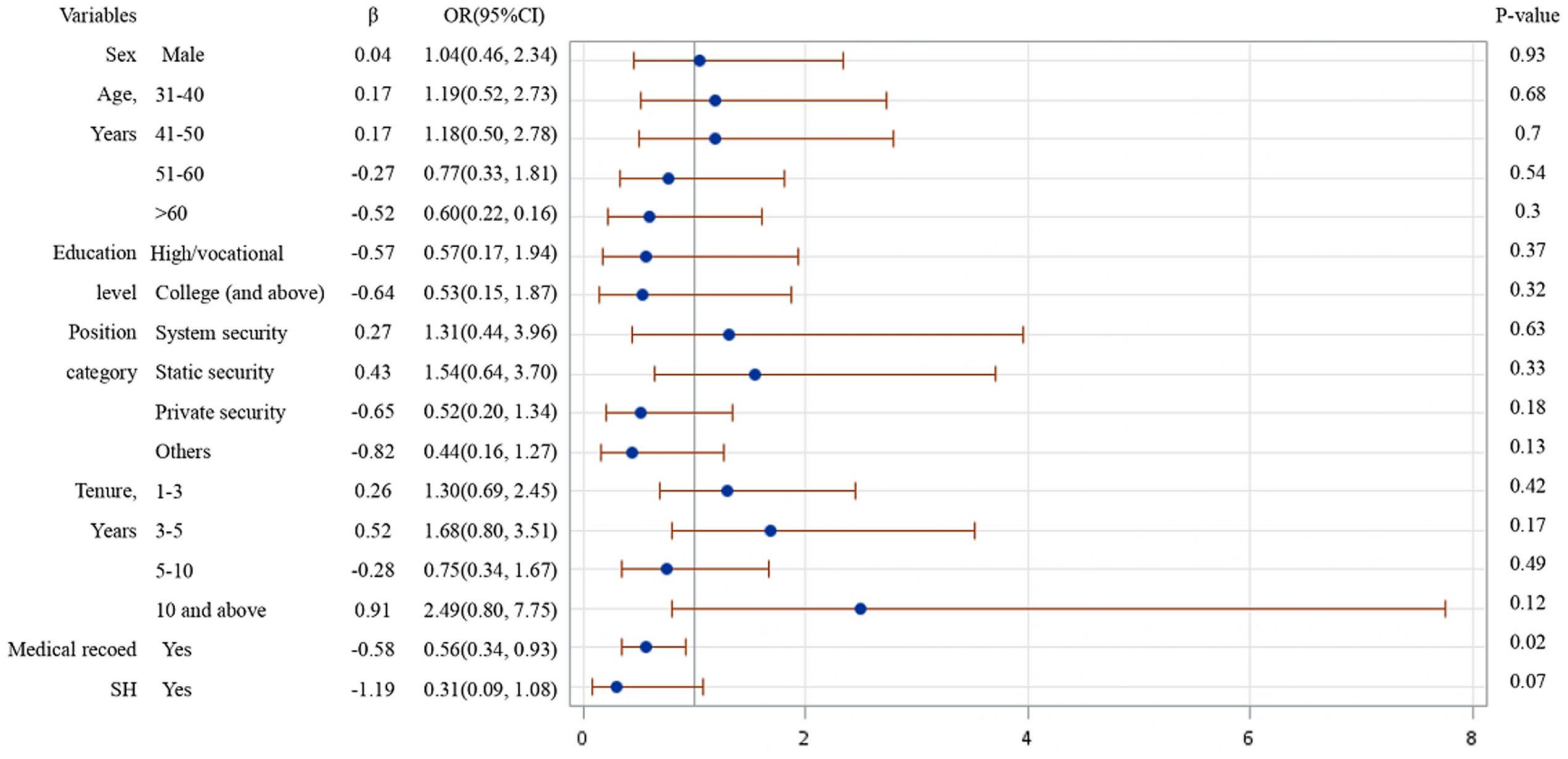
Logistic regression analysis on risk factors affecting MPH of participants experienced SH.

From the results of logistical regression in Figures 1–4, we found that after controlling potential confounding variables, including sex, age, education level, categories of job duties, and medical records, the experience (vs. non-experience) of any form of WPV was associated with poor MPH. The MPH of security guards was statistically related to PhV, VV, as well as PsV, and the ORs (95%CI) were 0.18 (0.08, 0.41), 0.20 (0.12, 0.34), and 0.09 (0.05, 0.19), respectively.

Table 4 analyzed the influence of WPV on somatization symptoms, anxiety and worry, depression, dysfunction of family relationship, and sleep disorders on the CHQ-12. The result shows that security guards who experienced (vs. non-experienced) WPV had both higher scores and medians in the various aspects in CHQ-12, indicating that WPV significantly affected somatization symptoms, anxiety and concern, depression, dysfunction of family relationship, and sleep disorders.

**Table 4:** If WPV has significant impact on physical symptoms, anxiety, and concern, depression and dysfunctional family relationships in CHQ-12.

Variables	Physical symptoms	Anxiety and concern	Depression and dysfunctional family relationships	Sleep disorders
	Medians (Q1–Q3)	Medians (Q1–Q3)	Medians (Q1–Q3)	Medians (Q1–Q3)
PhV				
Yes (*n* = 37)	0 (0–0)	1 (1–2)	1 (1–1)	0 (0–0)
No (*n* = 995)	0 (0–0)	1 (1–1)	1 (1–1)	0 (0–0)
*p* value	^***^	^***^	-	^**^
VV				
Yes (*n* = 110)	0 (0–1)	1 (1–2)	1 (1–1)	0 (0–1)
No (*n* = 922)	0 (0–0)	1 (1–1)	1 (1–1)	0 (0–0)
*p* value	^***^	^***^	^***^	^***^
PsV				
Yes (*n* = 42)	1 (0–1)	2 (1–2)	1 (1–2)	1 (0–1)
No (*n* = 990)	0 (0–0)	1 (1–1)	1 (1–1)	0 (0–0)
*p* value	^***^	^***^	^***^	^***^
SH				
Yes (*n* = 14)	0 (0–1)	1 (0–2)	1 (1–2)	0 (0–1)
No (*n* = 1,018)	0 (0–0)	1 (1–1)	1 (1–1)	0 (0–1)
*p* value	^**^	-	-	^*^

-, not significant; ^*^p < 0.05; ^**^p < 0.01; and ^***^p < 0.001.

The Cronbach’s *α* for the CHQ-12 questionnaire in this study was 0.87, The Cronbach’s *α* of PhV CHQ12, VV CHQ12, PsV CHQ12, and SH CHQ12 were 0.84, 0.83, 0.76, and 0.88 (Cronbach’s *α* >0.7 indicates high reliability). In addition, the Cronbach’s *α* of the security guards who experienced PhV, VV, PsV, and SH were 1.00, 0.57, 1.00, and 1.00 (0.30 < Cronbach’s Alpha <0.7 suggests moderate reliability).

## 4 Discussion

Our analysis revealed that the position category may have a role in WPV experiences. The majority of the participants in this study were static security guard (61.72%), who were our main study subject. During the COVID-19 pandemic, in addition to their regular duties, security guards undertook additional tasks, such as working with government health authorities for public awareness of COVID-19 virus development and monitoring, controlling, and providing the updates of residents in home quarantine to relevant agencies for pandemic prevention. Therefore, the security guards not only undertook heavier workloads due to the pandemic (7) but they also suffered deteriorating MPH resulting from WPV experience, social insecurity, and work stress (6).

This study found that the prevalence of any forms of WPV experienced by the participants during the pandemic was 13.18%. The most common was VV (54.19%), followed by PsV (20.69%). The prevalence of each type of WPV experienced by male and female security guards were PhV being 3.58 and 3.61%, VV being 10.43 and 13.25%, PsV being 4.00 and 4.82%, as well as SH being 0.95 and 6.02%. This result was close to the levels of WPV in the workplaces in Taiwan (8), showing that the most common type of WPV was VV, followed by PsV. It was consistent with the research conducted in other countries (16). One interesting finding was that, based on the research done in 2014, the prevalence of WPV against security guards in Taiwan was 16.71% (8), which was higher than our result. Moreover, a United States study on the violence against security guards in hospitals and ED dropped during the pandemic (6). Therefore, the prevalence of WPV against security guards might have decreased during the pandemic.

During the COVID-19 pandemic, when security guards performed epidemic preventive measures, the excessive waiting times and dissatisfaction with treatment often resulted in occurrences of VV and PsV against the guards. Their experiences with WPV significantly impact their MPH and led to burnout and turnover intention. In Taiwan, a study revealed the influence of psychosocial work conditions on the mental health of public health workers in 2023, pointing out that the employees who ever experienced WPV had a higher chance of intention to leave their jobs (31). It is important for the security industry to address and prevent WPV to protect the health and well-being of their staff. It could include implementing violence prevention programs, providing support and resources for employees who have experienced violence.

The main sources of WPV are from vertical, horizontal, and third-party perpetrators (21). According to our analysis, the main factors affecting MPH of the participants were VV and PsV from the third-party perpetrators, with community residents and property owners constituting the majority. Its main reason was the lack of effective communication. After experiencing VV and PsV, most of the victims would seek colleagues’ assistance. A study conducted in Italy addressed that high social support was a protective factor for healthcare sector workers, preventing them from third-party violence (32). Although the examined work sectors were different, the WPV perpetrators both came from the third party, which required further study to explore the relevant risk factors.

Table 1 addressed that the male and female security guards experienced different forms of WPV in different levels. The percentages of the WPV experienced by male and female were: PhV (3.52 and 3.61%), VV (10.43 and 13.25%), PsV (4 and 4.82%), and SH (0.95 and 6.02%). Furthermore, by performing Chi-square test, the result addressed that there was no statistical significance between gender differentiation and experiencing any forms of WPV, PhV, VV, and PsV (*p* = 0.1693, 0.9881, 0.4245, 0.7690). Nevertheless, the relation between gender differentiation and experiencing SH at the workplace showed statistically significance (*p* = 0.0034). A study in the United States on WPV among physicians demonstrated that there were gender differences in experiencing workplace violence. The result showed that males and females reported similar frequency of physical abuse (2.2%), while females (33%) were more likely to experience verbal violence than males (28%). Moreover, sexual harassment was reported by 19.9% of women and 3.9% of men. Our research revealed that there was a higher prevalence of VV and SH occurred on the female security guards than males, which was correspondent in the security industry and surgical residency (33).

Our analysis reveals that work stress was related to verbal violence, but not to other types of violence. In addition, the security guards who had experienced WPV had higher risk of having poorer MPH than those who had not experienced WPV. This result was consistent with an investigation on the association between the WPV violence and the health status of Taiwanese employees (8), denoting WPV led to poor health. Furthermore, a longitudinal analysis on Swiss population of professional caregivers suggested that psychophysiological stress response is primarily associated with combined verbal and physical aggression (34). It was proved that health of the professions in both service or healthcare sectors were adversely affected due to WPV.

Furthermore, our analysis discovered that WPV experienced by security guards was significantly associated with somatization symptoms, anxiety and concerns, depression and dysfunction of family relationships, and sleep disorders. At the same time, their experience of PhV, VV, or PsV was significantly correlated with the scores of CHQ-12. In other words, the security guards who experienced PhV, VV, or PsV had poorer MPH. A Taiwanese study on workers of public health agencies found a significant correlation between high prevalence of workplace violence in and increased risk of mental disorders (31). A Japan study in 2022 also revealed that public healthcare workers (HCWs) who experienced increased harassment could have a higher risk of depression and anxiety (35). The research showed that, compared to other occupations, the negative impacts of WPV on mental health were more severe for both security guards and HCWs.

The negative impact of WPV is evident and requires attention of nations to enact laws and policies, endeavoring to ensure the rights and physical and mental health of workers. For instance, in 2020, the Italian Parliament passed a law to impose harsher punishments for acts of violence against medical professionals, particularly when there was an injury associated (36). In Taiwan, the Taiwanese Government revised Occupational Safety and Health Act in 2014 to prevent physical or mental unlawful infringement caused by the actions of others during the execution of job duties. These legal changes have demonstrated that relevant government authorities committed to addressing the issues of WPV and the potential health impact. Because security guards are in a high-risk occupation susceptible to WPV, as well as workplace physical and psychological harm (37), their health and safety should be taken into special consideration regarding legal enforcement in the workplaces. Furthermore, future research on determining the relationship between WPV and MPH of security guards is also recommended.

The limitations and future studies should be highlighted. A cross-sectional survey was conducted on the security guards and data were collected from purposive sampling. First, it implied the uncertainty of the causes and sequence of WPV. Second, the causal relationship between WPV experience and medical records could not be assessed. Third, there might be sampling and selection biases, meaning the study might have introduced sample selection bias and memory recall bias of the participants. Fourth, the survey result might be affected by the subjective consciousness of participants, which impacted the self-reported data. Fifth, in this study lacked the analysis on gender differentiation due to the gender distribution of the security industry. Therefore, we could not collect sufficient data of the females.

## 5 Conclusion

This study is the first exploration of the impact of WPV on the MPH of security guards during the COVD-19 pandemic. It revealed that during the pandemic, there were statistically significant relationships among PhV, PsV, and VV, which adversely affected the MPH of security guards. The primary WPV perpetrators against static security guards were from the third party, including community residents and property owners. The factors contributing to WPV included the lack of effective communication, dissatisfaction with treatment and service attitude, excessive waiting times, and, work stress, which further resulted in burnout, turnover intention, and poor MPH. In the meanwhile, the results of this study can provide a reference for government authorities, academic institutions, and relevant agencies to enact laws and policies to prevent workplace violence and to protect the physical and mental health of security guards.

## Data availability statement

The original contributions presented in the study are included in the article/Supplementary material, further inquiries can be directed to the corresponding authors.

## Ethics statement

This research was approved by the Behavioral and Social Science Research Ethics Committee, China Medical University Hospital (Identifier: CMUH110-REC1-124). The participants provided their written informed consent to participate in this study.

## Author contributions

Y-HL: Conceptualization, Formal analysis, Methodology, Writing – original draft. Y-HW: Conceptualization, Data curation, Project administration, Visualization. C-YC: Writing – review & editing. PC-T: Visualization, Writing – review & editing. T-HL: Formal analysis, Methodology, Visualization. C-YL: Visualization. All authors have read and agreed to the published version of the manuscript.
